# A Technical Report for Definitive Treatment of Uveal Melanoma With Stereotactic Radiosurgery and Retrobulbar Anesthesia

**DOI:** 10.7759/cureus.86296

**Published:** 2025-06-18

**Authors:** Austin D Williams, Milton Boniuk, Daniel Hamstra, Ryan T Wallace, Larry S Carpenter

**Affiliations:** 1 Department of Radiation Oncology, Baylor College of Medicine, Houston, USA; 2 Department of Ophthalmology, Baylor College of Medicine, Houston, USA

**Keywords:** eye cancer, linac based radiosurgery, malignant uveal melanoma, organ sparing treatment, preservation of vision, retrobulbar anesthesia, review of literature, single fraction stereotactic radiosurgery, technical report

## Abstract

Uveal melanoma (UM) is the most common primary intraocular malignancy, traditionally managed with episcleral plaque brachytherapy or enucleation. Single-fraction stereotactic radiosurgery (SRS) using a linear accelerator (LINAC) is an emerging alternative offering high precision and eye preservation. However, existing SRS/stereotactic radiation therapy (SRT) techniques often rely on mechanical immobilization or patient-maintained fixation. To the best of our knowledge, this is the first report of single-fraction, LINAC-based, frameless robotic SRS with retro- and peribulbar anesthesia for ocular immobilization from the United States. Additionally, we review the literature to assess local control, enucleation rates, and treatment-related toxicity of SRS/SRT for UM.

Patients diagnosed with UM at our institution were evaluated by a multidisciplinary team of radiation oncologists and ophthalmologists. A thermoplastic mask and retro- and peribulbar anesthesia were used for ocular immobilization. Treatment planning involved computed tomography (CT) and magnetic resonance imaging (MRI) fusion for target delineation. Gross tumor volume (GTV) encompassed the entire tumor, with a 2 mm planning target volume (PTV) expansion and no clinical target volume (CTV). Treatment was delivered using the CyberKnife® system (Accuray Incorporated, Sunnyvale, California, United States) in a single 20 Gy fraction at the 80% isodose line, with real-time imaging for precise radiation delivery. Patients were monitored post-treatment for acute complications, with follow-up assessments of local tumor control, eye retention, and treatment-related toxicity. Additionally, we conducted a literature review of SRS/SRT studies, collecting data on patient numbers, tumor size, radiation regimens, immobilization techniques, and clinical outcomes, including local control, complications, and eye retention rates.

This study demonstrates the feasibility of single-fraction, frameless LINAC-based SRS using CyberKnife with retro- and peribulbar anesthesia as an effective, patient-friendly alternative for treating UM. Patients with medium to large tumors are excellent candidates, even with a single 20 Gy fraction. This technique eliminates the need for mechanical eye immobilization while maintaining tumor control rates comparable to established modalities with potentially improved patient comfort. We aim to further evaluate our cohort’s long-term patient outcomes, including local control, vision preservation, and late toxicities.

## Introduction

Uveal melanoma (UM) is the most common primary intraocular malignancy, typically identified through vision changes or during routine screenings in adults aged 50-70 years [1]. Surgical treatment would typically involve removal of the entire eye; however, for functional and cosmetic reasons, many patients would like to avoid enucleation. The Collaborative Ocular Melanoma Study (COMS), a multicenter randomized trial, compared Iodine (I)-125 brachytherapy to enucleation in 1,317 patients and found no significant mortality differences after 12 years, thus supporting organ-sparing approaches [2]. Advances in radiotherapeutic (RT) techniques over the last four decades have reduced enucleations and preserved patients’ vision without compromising the ~80% five-year relative survival rate [3].

For early-stage, localized UM, RT options include ophthalmic brachytherapy, charged particle therapy, and stereotactic radiosurgery (SRS), which often may be chosen based on tumor thickness and proximity to critical structures. Research to improve treatment efficiency and patient comfort is ongoing. Ophthalmic brachytherapy is the most performed and well-studied technique. However, it commonly requires two procedures under general anesthesia, involving the placement and subsequent removal of radioactive episcleral plaque. COMS-style plaques are also limited to medium-sized tumors; however, larger lesions can sometimes be treated with a two-part staged treatment [4]. Furthermore, they suffer from dose heterogeneity [5]. Charged particle therapy has been used to treat UM, but accessibility remains severely limited [6]. This modality also requires dual procedures to place and remove radiographic tantalum ring fiducial markers.

Stereotactic radiation therapy (SRT) focuses high RT doses in one or more fractions to a precise target volume. Single-session frame-based Gamma Knife® (Elekta AB, Stockholm, Sweden) radiosurgery has demonstrated high local control (91-93%) and eye retention rates (86-90%) in patients with UM [7]. However, the requisite stereotactic head frame and mechanical eye immobilization techniques can be burdensome for patients and providers [8]. An attractive alternative consists of single-session frameless linear accelerator (LINAC)-based SRT using the CyberKnife® system (Accuray Incorporated, Sunnyvale, California, United States), computed tomography (CT)-planning, and retrobulbar anesthesia, which has shown comparable efficacy to ophthalmic brachytherapy and proton therapy [9].

This report describes the first-known United States application of LINAC-based, frameless, robotic, single-fraction SRS using retrobulbar anesthesia at Baylor Catholic Health Initiatives (CHI) in Houston, Texas.

## Technical report

Study design

This is a technical report of single-session stereotactic radiosurgery for the treatment of UM using CyberKnife and retrobulbar anesthesia. Our study protocol (H-57273: LINAC-Based Cyberknife for the Treatment of Uveal Melanoma) was approved by the Baylor College of Medicine Institutional Review Board.

Diagnosis and CT-simulation

**Figure 1:**
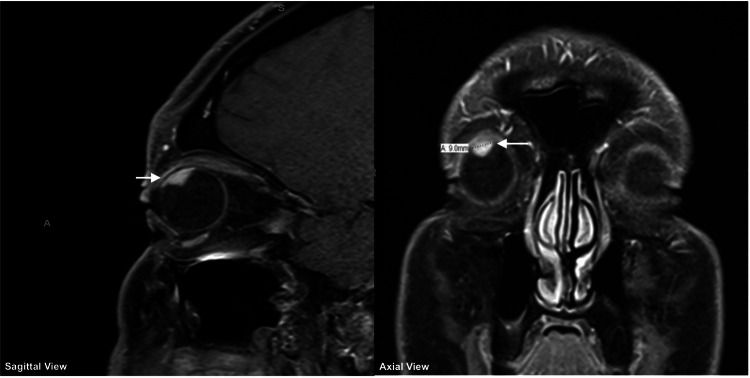
MRI of patient with right 9 mm (COMS-medium) uveal melanoma (sagittal, axial views) COMS: Collaborative Ocular Melanoma Study

Patients with suspected UM undergo evaluation by a physician with experience in ocular oncology. They are referred to a medical oncologist for staging and evaluation for metastasis. Fundoscopic imaging alongside CT and/or magnetic resonance imaging (MRI) assists in RT planning (Figure 1). Following informed consent for RT, patients undergo CT-based simulation in the treatment position, including a custom-fit thermoplastic (Aquaplast) mask. 

Retrobulbar and peribulbar blocks for ocular akinesia

On the day of treatment, the ophthalmologist visits the RT facility to deliver a retrobulbar block with the goal of complete eye immobilization. This may even be performed while the patient lies on the CT-simulation table. No rectus muscle sutures are required using retro and peribulbar blocks. A mixture of 2% lidocaine without epinephrine and 0.5% bupivacaine is used for local anesthesia, combined with hyaluronidase 25 IU/mL per provider preference. Using retro and peribulbar anesthesia, along with efficient dose planning and delivery, most patients may be discharged within four hours of administering local anesthetic. An experienced ophthalmologist should remain present to monitor patients for retrobulbar hemorrhage and globe perforation. 

A standard retrobulbar block consists of providing anesthetic in the intraconal space, which comprehensively blocks cranial nerves (CN) II, III, and VI, as well as the ciliary nerves and ganglion. With the patient’s gaze fixed in a neutral position, a 23- or 25-gauge, 1.5-inch needle is positioned perpendicularly at the junction of the lateral one-third and medial two-thirds of the lower eyelid. The needle is slowly advanced through the orbital septum, then redirected 30-45º superiorly and slowly advanced through the intermuscular septum about 1 inch. After aspirating to confirm the needle tip is not in an intravascular space, 2-5 mL of the local anesthetic mixture is slowly injected. Clinically, slight proptosis of the globe will result after injection and resolve over several minutes as the block solution diffuses either spontaneously or assisted by light ocular massage. The eye may be examined to confirm ocular akinesia after five minutes.

A peribulbar block consists of providing an anesthetic in the superior and/or inferior extraconal spaces. There are multiple variations of this technique, some of which can block the superior oblique supplied by cranial nerve IV, which is largely spared by retrobulbar anesthesia. In a traditional peribulbar block, a shorter 0.5-1 inch needle is positioned through the superior septum (depending on provider preference, this is directly at 12’ or slightly more nasal to 12’ to target the superior oblique). The needle is slowly advanced parallel to the globe, and 3-4 mL of local anesthetic mixture is slowly injected into the superior extraconal space. This is followed by a similar injection and volume inferiorly. Other techniques include transcaruncular or even trochlear approaches to further target the superior oblique. Gentle pressure is applied to the eye for 10-15 minutes, and the eye is examined to confirm ocular akinesia. 

Treatment planning

**Figure 2:**
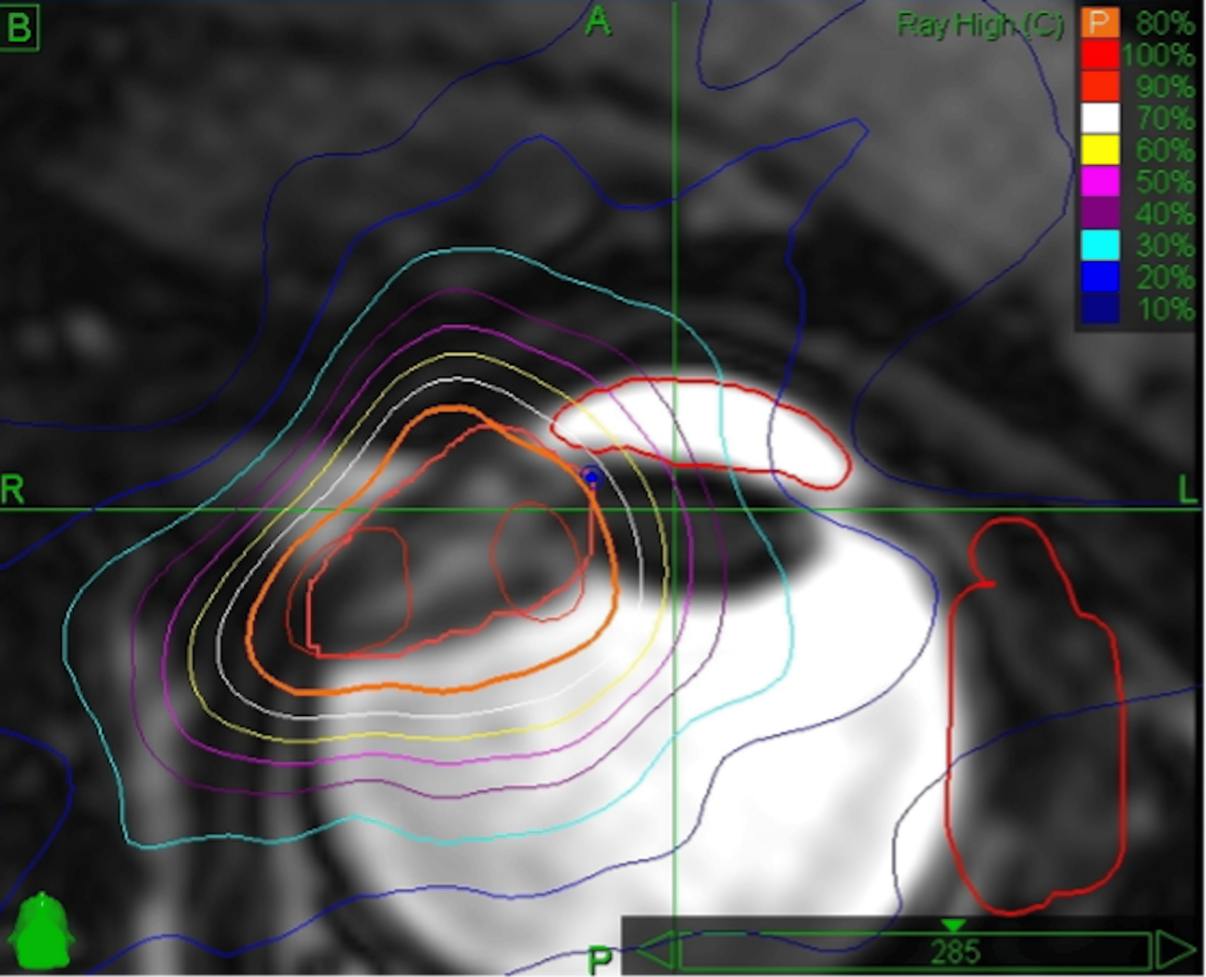
Left eye uveal melanoma with contours traced on CT/MRI fusion; 20 Gy at 80% isodose is prescribed to the PTV (orange) (GTV + 2 mm) with no CTV. PTV: planning target volume; GTV: gross tumor volume; CTV: clinical target volume

After achieving effective extraocular muscle paralysis, a non-contrast MRI is obtained for one or two sequences, including thin-slice volumetric non-contrast axial fat-suppressed (FIESTA) sequences. The MRI is fused to the previously acquired CT images for immediate planning of target and avoidant structures, including the optic nerve, cornea, and lacrimal gland (Figure 2). The full thickness of the sclera is included in the target volume. A 2 mm planning target volume (PTV) is added to the gross tumor volume (GTV) with no clinical target volume (CTV) used. A single fraction of external beam radiation to a prescription dose of 20 Gy at 80% isodose is administered to the PTV with CT/MRI-fused guidance. 

Planning and delivery were performed using the CyberKnife® system, which uses a compact 6 MV linear accelerator attached to an industrial robot and real-time kV imaging. These imagers are used for near real-time fusion of skull radiographs with the digitally reconstructed radiographs from the planning CT. 

## Discussion

To our knowledge, this represents the first report from the United States of a single-fraction radiosurgical approach for treating UM, employing a LINAC-based platform (CyberKnife) and a landmark-guided retrobulbar anesthetic block to achieve ocular akinesia. Our technique leverages key advantages of frameless SRS, including outpatient feasibility and rapid treatment delivery without the need for operating room access or multiple sessions, as is required with episcleral plaques and proton beam therapy. Moreover, this differs from existing techniques that rely on general anesthesia, patient-maintained fixation, or invasive mechanical immobilization using suction devices or suture loops.

Most commonly, patients with medium-sized tumors (5-7 mm diameter) are treated using conventional COMS-style episcleral plaques with I-125 of Ruthenium (R)-106. Limitations include a maximum apical height of <10 mm, a basal diameter of 16 mm, and a 90º angle from the optic disc apex [4,10]. Modified plaque designs aim to spare adjacent structures and maintain a 2 mm margin beyond the tumor, as recommended by the American Brachytherapy Society. However, dose heterogeneity remains a challenge, with surface doses exceeding the apex by 2.5-8 times [5,11]. Nevertheless, in a small sub-cohort of 13 patients, Miguel et al. demonstrated that five-year local control rates of more than 80% may be achievable with large (<10 mm) tumors [12]. 

CyberKnife radiosurgery has been used internationally for medium-to-large UM, with excellent dose homogeneity when paired with thermoplastic masks and retrobulbar anesthetic blocks [13]. The University of Munich group has the most extensive experience, using frameless, image-guided robotic radiosurgery with peribulbar anesthesia in a streamlined, single-session workflow. Their five-year outcomes include local control of 70.8%, eye retention of 73.0%, and functional vision preservation in 31% of patients [9]. A follow-up study involving 594 patients showed improved five-year local control of 87.5% and eye retention of 81.7%, with a median dose of 20 Gy (Table 1) [14].

**Table 1:** Comparing studies on LINAC-based SRT and hypofractionated SRS *Collaborative Ocular Melanoma Study (COMS) size classification: S=small, M=medium, L=large Fx: fraction; LINAC: linear-accelerator; SRS: stereotactic radiosurgery; SRT: stereotactic radiation therapy

Author, Year	Location	Number of Patients	Tumor Size* (percentage of patients)	Radiation Treatment	Dose	Immobilization Technique	Local Control (percentage of patients at n-years)	Treatment-Associated Complications (percentage of patients)	Eye Retention (percentage of patients)
Eibl-Linder, 2016 [9]	Munich, Germany	217	S 3.3%; M 66.9%; L 29.8%	Single-fraction SRS	20 Gy median	Retro-/Peribulbar Block	87.4% at 3 years; 70.8% at 5 years	Retinopathy 13.4%; Neovascular glaucoma 15.2%; Hemorrhage 12.0%	86.7% at 3 years; 73.0% at 5 years
Cappelli, 2025 [15]	Philadelphia, United States	23	-	5-fraction SRT	10 Gy/fx	Patient-maintained fixation	85.6% at 3 years	Papillopathy 39%; Retinopathy 22%; Maculopathy 9%; Optic atrophy 9%	91.3% at 3 years
Dunavoelgyi, 2011/2012 [16,17]	Vienna, Austria	212	S 8.0%; M 89.2%; L 2.8%	5-fraction SRT	10-14 Gy/fx	Mechanical fixation	95.9% at 5 years; 92.6% at 10 years	Retinopathy 53.8%; Papillopathy 50.5%; Cataract 41.0%; Neovascular glaucoma 21.7%	81.6% at 10 years

Other LINAC-based techniques have been described in the United States, though none using a single fraction or anesthetic block. Cappelli et al. (Thomas Jefferson) treated 23 patients over five fractions using a custom stereotactic localization box without anesthesia or mechanical restraint [15]. They reported a three-year local control of 85.6% and eye retention of 91.3%, with complication rates including radiation papillopathy (39%) and retinopathy (22%). Dunavoelgyi et al. (University of Vienna) reported a similar five-fraction regimen with excellent long-term control (92.9% after 10 years) but higher rates of radiation-associated toxicity, including retinopathy (53.8%) and neovascular glaucoma (21.7%) [16,17].

Direct comparisons of complication rates remain limited due to the variation in treatment plans, tumor sizes, and follow-up. Across studies, commonly reported toxicities include cataracts, radiation retinopathy and maculopathy (9-81%), optic neuropathy (39%), and neovascular glaucoma (17.3-45%), the latter of which may necessitate enucleation [15,18,19]. Retinopathy remains the leading cause of permanent vision loss, emphasizing the need for annual surveillance, especially in patients with large-diameter tumors [9].

Krema et al. directly compared I-125 brachytherapy and SRT for 94 patients with juxtapapillary tumors and found that SRT delivered significantly higher radiation doses to the optic disc and adjacent retina at a 5 mm distance from the tumor margin [20]. These patients, who lacked mechanical or anesthetic-induced akinesia, experienced increased rates of neovascular glaucoma, papillopathy, and retinopathy, raising concerns about external beam approaches for juxtapapillary lesions [19]. Still, there are locations of the eye where episcleral plaques cannot be used and where the potential for LINAC-based SRS is clearer.

Gamma Knife also provides a single-fraction treatment for medium and large UM but requires invasive stereotactic headframes and ocular muscle sutures for immobilization [7]. Moreover, the prescribed dose typically represents 50% of the maximum delivered dose, introducing inhomogeneity that involves the sclera. In contrast, the CyberKnife approach described here offers dose homogeneity without invasive fixation, potentially improving workflow, patient experience, and target coverage.

While preliminary results from our experience treating 21 patients in Houston, Texas, United States, are promising, we have deliberately excluded original outcome data from this report. We are currently compiling a dedicated case series to formally analyze patient outcomes, including local control, complications, and visual function. This technical report provides the rationale and context for our approach and will complement future outcome-based studies using this modality.

Lastly, ongoing advances in intensity-modulated spot-scanning proton therapy continue to improve dose conformity, though access remains limited. In contrast, LINAC-based platforms, such as CyberKnife, are more widely available and affordable. Our approach offers a practical and scalable solution to expand access to effective UM treatment in diverse clinical settings.

## Conclusions

To our knowledge, this is the first report from the United States of single-fraction LINAC-based radiosurgery using a retrobulbar anesthetic block to achieve ocular akinesia. This approach is appropriate for medium-to-large ocular melanomas, particularly those malpositioned for conventional episcleral plaque brachytherapy. While SRS for UM shares common risks of radiation-associated complications, including cataract formation, radiation retinopathy, and neovascular glaucoma, it has the potential to achieve excellent local control and organ preservation rates. We look forward to sharing outcome data in subsequent publications.
